# The alpha-2A adrenoceptor agonist guanfacine improves sustained attention and reduces overactivity and impulsiveness in an animal model of Attention-Deficit/Hyperactivity Disorder (ADHD)

**DOI:** 10.1186/1744-9081-2-41

**Published:** 2006-12-15

**Authors:** Terje Sagvolden

**Affiliations:** 1 Institute of Basic Medical Sciences, Department of Physiology, University of Oslo, P.O. Box 1103 Blindern, NO-0317 Oslo, Norway

## Abstract

**Background:**

ADHD is currently defined as a cognitive/behavioral developmental disorder where all clinical criteria are behavioral. Overactivity, impulsiveness, and inattentiveness are presently regarded as the main clinical symptoms. There is no biological marker, but there is considerable evidence to suggest that ADHD behavior is associated with poor dopaminergic and noradrenergic modulation of neuronal circuits that involve the frontal lobes. The best validated animal model of ADHD, the Spontaneously Hypertensive Rat (SHR), shows pronounced overactivity, impulsiveness, and deficient sustained attention. While dopamine release is decreased in SHR prefrontal cortex, norepinephrine concentrations are elevated. The noradrenergic system appears to be hyperactive as a result of impaired alpha-2A adrenoceptor regulation. Thus, the present study tested behavioral effects of the centrally acting alpha-2A adrenoceptor agonist guanfacine on SHR behavior.

**Methods:**

The present study tested behavioral effects of guanfacine at doses of 0.075, 0.15, 0.30 and 0.60 mg base/kg i.p. in both male SHRs and their controls, the Wistar Kyoto rat (WKY). ADHD-like behavior was tested with a visual discrimination task measuring overactivity, impulsiveness and inattentiveness.

**Results:**

The striking impulsiveness, overactivity, and reduced sustained attention during baseline conditions in the SHR improved by treatment with guanfacine. The most pronounced improvement in SHR behavior was seen following the two highest doses (0.3 and 0.6 mg/kg) of guanfacine when SHR behaviors virtually normalized. The positive effects of the drug were most marked towards the end of the session.

**Conclusion:**

The results indicate that guanfacine improved poor noradrenergic modulation of neuronal circuits that involve the frontal lobes in an animal model of ADHD. The present results support the beneficial effects of guanfacine on ADHD behavior reported clinically and experimentally in primate models of frontal function. It is likely that guanfacine improved prefrontal functions in the SHR. It cannot be concluded, however, that the effects of the drug are mediated solely by norepinephrine.

## Background

Attention-deficit/hyperactivity disorder (ADHD) is currently defined as a cognitive developmental disorder where all clinical criteria are behavioral [1]. Overactivity, impulsiveness, and inattentiveness are presently regarded as the main clinical symptoms.

There have been many attempts to explain the origins of ADHD symptoms. A dual-process model [2-5] suggests that less efficient reinforcement processes and deficient extinction of previously reinforced behavior are fundamental to the problems described as response inhibition [6] and poor executive functions [7].

ADHD is highly heritable and the genetic and neurobiological causes are likely to reside in brain catecholaminergic systems (for a review see [4]). Most likely, ADHD symptoms are associated with dysregulation of dopaminergic and noradrenergic modulation of neuronal circuits that involve the frontal lobes [8,9]. Prefrontal cortical neurons are able to hold information relevant for the next behavior [10]. Such information may be weakened by dysregulated dopaminergic and noradrenergic systems in ADHD causing the deficient working memory [11] and the need for immediacy of reinforcement [4].

Although stimulants are the drugs of choice in the treatment of ADHD [12,13], more than 10% of children and adults with ADHD do not respond to stimulants or are unable to tolerate the side effects [13]. Consequently, there is a need for alternatives to stimulant medication.

Guanfacine has been used as a medication for ADHD [12,13] although the precise mechanism of action of guanfacine in ADHD is unknown. Guanfacine appears to mimic the effect of norepinephrine at alpha-2A adrenoceptors, improving prefrontal cortical cognitive functions at the cellular and behavioral levels (for a review see [14]). These apparent effects are also reflected in imaging studies demonstrating increased prefrontal cortical blood flow following administration of guanfacine [15].

The spontaneously hypertensive rat (SHR) is the best validated animal model of ADHD. These rats show hyperactivity, impulsiveness and deficits in sustained attention [9,16-18]. The control strain is usually the Wistar Kyoto Rat (WKY) as this rat is the progenitor strain and its behavior is closely similar to that of other strains when tested in operant tasks [17].

Dopamine release is decreased in SHR prefrontal cortex and norepinephrine concentrations are elevated [19,20]. The noradrenergic system appears to be hyperactive as a result of impaired alpha-2A adrenoceptor regulation [9]. Thus, the aim of the current study was to investigate behavioral effects of a wide dose range of the alpha-2A adrenoceptor agonist guanfacine hydrochloride in the SHR animal model of ADHD.

## Methods

### Subjects

A total number of 32 male rats, 16 SHR and 16 WKY, participated in this study. At the start of testing following 8 days acclimatization, the rats were 4 wk old and experimentally naïve at the start of study. Young rats were required, as ADHD primarily is a child and adolescent disorder. The rats were obtained from Charles River Italy (SHR/Crl Ico). At the University of Oslo, the rats were housed individually in 41 × 25 × 25 (height) cm transparent cages and had free access to food (Special Diet Services, Witham, Essex, UK.).

The rats had access to water at all times before the habituation session. However, after completing the habituation session, the rats were deprived of water for 21 hr a day. The rats received water as reinforcers during the experimental session and had free access to water for 90 min after the experimental session. The temperature in the housing area was ~22°C. The light in the housing area was on from 0700 to 1900 hours. The behavioral training took place between 1000 and 1330 hours seven days a week.

### Behavioral apparatus

Sixteen Campden Instruments operant chambers were used in the study. The animal working space in eight of the chambers was 25 × 25 × 30 (height) cm and 25 × 25 × 20 (height) cm in the other eight chambers. The 2.8-W house light and a fan producing a low masking noise were on during the entire experimental session.

During training sessions, either one or both retractable levers were used (below). A 2.8-W cue light was located above each lever. The rats' response consisted of pressing one of the levers with a dead weight of at least 3 g to activate a micro-switch. The reinforcers (0.01 ml tap water) were delivered by a liquid dipper located in a small recessed cubicle with a 2.8-W cue light lit up when a reinforcer was presented. A 7 × 5 cm transparent plastic lid separated the cubicle from the rat's working space. The rat could easily open the lid with a light push with the nose or paw. Each chamber was ventilated and placed in a sound-resistant outer housing. A computer and an online system (SPIDER, Paul Fray, Ltd., UK) recorded the behavior and scheduled reinforcers (drops of water).

Before the initiation of the study, the rats were assigned a chamber (1 through 16) and time of testing (1000 or 1200 hours) in a randomized and balanced way. The rat was returned to its living cage after each session and immediately given free access to water for 90 min. All animals were run seven days a week.

### Response acquisition

The training period started with a single 30-min habituation session. During the habituation session, the lid between the working space and the reinforcement cubicle was kept open. No lever was present, no cue light above any lever was lit and water was not delivered. The house light was on. Following completion of the habituation session the rats were deprived of water for 21 hr a day; this is a moderate, but sufficient deprivation for motivating the animal.

The habituation session was followed by two 30-min magazine training sessions. The lid was taped open, no levers were present, and the house light was on, but the cue lights above the levers were not lit. The computer delivered water on the average every 10 s independent of the rat's behavior (a variable-time schedule). Each water delivery was accompanied by the turning on of the cue light in the small recessed cubicle.

In the next four sessions, the rat was trained to open the lid to gain access to the water. The lid was not taped open, no levers were present and the lights above the levers were not activated. The house light was on. Each lid opening was followed by a presentation of a single drop of water. The cue light in the recessed cubicle was turned on when the water was presented.

During the subsequent three sessions, lever responding was shaped by the method of successive approximations [21]. During the first two of these sessions, the rats learned to press the left lever in order to receive a reinforcer after every press. The cue light above the left lever was now lit. The house light was on. The right lever was retracted into the wall and the light above the right lever was not lit. On the third session, the right lever was activated and the left lever retracted. During this session the light above the right lever was activated, but the light above the left lever was turned off. The house light was on. Following this shaping procedure the animal had acquired the appropriate behavior. From now on, both levers were present. The light above the levers shifted randomly. The light showed the rat which lever it had to press to receive a reinforcer ("correct lever"). The next five sessions lasted for 30 min and the reinforcers were delivered following every correct lever press. The rat received the reinforcer immediately after pressing the correct lever. The cue light in the recessed cubicle was turned on when the water was presented. A concurrent extinction (EXT) schedule was present on the wrong lever.

### Final schedule

From session 16 on until the study was finished, a 180-s Random-Interval (RI) schedule was in effect on the correct lever. A concurrent extinction (EXT) schedule was present on the wrong lever. A reinforcement schedule is called multiple when two (or more) schedules are run and each of these is signaled. Thus, a multiple Random-Interval/Extinction (mult RI EXT) schedule was applied for testing effects of the drugs in the present study.

During the final multiple Random-Interval/Extinction schedule, reinforcers were delivered on average every 180s. There was neither any external stimulus signaling that a reinforcer was programmed according to the RI schedule, nor any external stimulus signaling the time since the last response.

### Behavioral measures

Each session was divided into five 18-min parts ("segments") in order to monitor intra-session changes in the behavior. For each segment, each lever press was recorded as a function of time since last response (inter-response time, IRT). Further, the number of reinforcers delivered was recorded for each segment.

The total number of lever presses is an expression of the general activity level and therefore a measure of degree of *overactivity*. The percentage of correct lever choices of the total number of lever presses when the reinforcers are delivered infrequently is a measure of *sustained attention*. The number of responses with short IRTs (< 0.67 s) is a measure of degree of *impulsiveness *("cannot hold back a response even when one knows it is an unnecessary one").

### Drug administration

In order to habituate the rats to injections and check drug effects, the rats were given a single 0.30 mg/kg injection of guanfacine on session 44, i.e., 5 days before the first treatment day. Administration of the drugs started at session 49, following behavioral stabilization. The effects of guanfacine hydrochloride were compared with vehicle. Each rat was dosed intraperitoneally (i.p.) at a dose volume of 1 ml/1 kg body weight of the animal ~30 min before testing, with either vehicle (saline) or drug. Drugs were administered every 3rd day. All rats received all doses according to a balanced design.

*Doses *were 0.075, 0.15, 0.3, and 0.6 mg/kg guanfacine, and were calculated as the weight of base using a conversion factor of 1.15 mg hydrochloride salt as equivalent to 1.0 mg base. Dosing solutions were prepared as a solution in physiological saline. A stock solution, 0.6 mg/ml guanfacine, was prepared just prior to every second drug session, i.e., every 6^th ^weekday and kept at +4°C when not in use. Dilutions were made just prior to dosing.

### Data management and statistical procedures

The mean behavior is regarded as the drug response, and dose-response curves are plotted for each drug and strain. The data are processed by univariate analyses of variance (ANOVAs) with the Statistica 5.5 program [22]. Within-subject variables are dose and within-session segment. Strain is a between-subject variable. Univariate ANOVAs with Greenhouse-Geisser corrections of the univariate *Fs *and the multivariate test Rao R Form 2 are reported in order to correct for repeated measures within subjects. The no-injection sessions are not included in the statistical analyses, but are shown in the figures in order to facilitate evaluation of drug effects. T-tests are used to follow up the ANOVAs.

## Results

### General

SHRs showed pronounced impulsiveness, overactivity and decreased sustained attention. These behaviors improved and virtually normalized following the highest doses (0.3 and 0.6 mg/kg) of guanfacine.

### Acquisition

**Figure 1:**
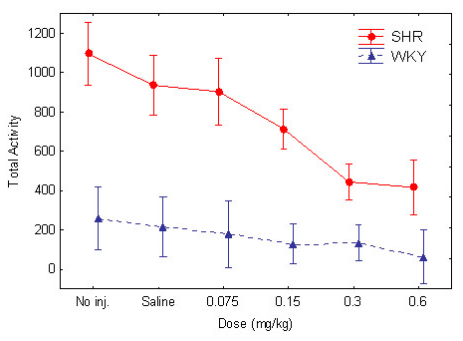
Effect of the different guanfacine doses on total number of lever presses in SHR and WKY. Means ± 95% confidence intervals.

As is the case in children with ADHD, the symptoms developed with time, but differently for the different behaviors. The final schedule was installed on session 16. A pronounced overactivity was seen in SHR from session 18 onwards (Figure 1, see also Additional files 1 and 2). SHR impulsiveness, responding within 0.67 s since the previous lever press (although such a lever press was almost never reinforced), continued to increase in the SHR throughout the entire study (not shown). This measure was accompanied by increased variability over days during the course of the experiment, something that is typical in ADHD. In order to obtain more equal variances as required by the ANOVAs, impulsiveness was subjected to a lg10-transformation. These transformations were used for evaluating drug effects.

#### Hyperactivity

**Figure 2:**
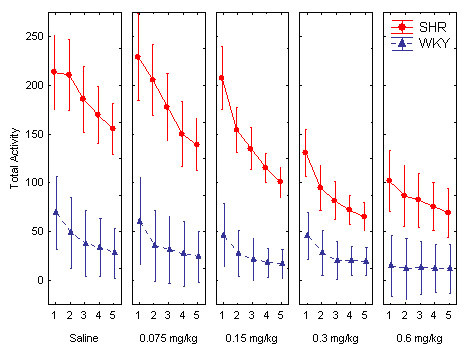
Effect of the different guanfacine doses on total number of lever presses across the five 18-min within-session segments in SHR and WKY. Means ± 95% confidence intervals.

There was a pronounced hyperactivity and good behavioral separation between the two strains (Figures 1 and 2). The pronounced SHR overactivity was reduced by guanfacine. Following the highest doses of the drug, 0.3 and 0.6 mg/kg, the general activity level of the SHRs approached that of the WKY controls. The ANOVA showed main effects of strain, *F(1, 30) = 57.84, p < 0.001*; dose, Greenhouse-Geisser ε = *0.65, F(2.6, 78.5) = 20.3, p < 0.001*; and within-session segment, Greenhouse-Geisser ε = *0.38, F(1.5, 55.6) = 49.8, p < 0.001*, showing that SHRs were significantly different from WKYs on this measure. There was also a Strain x Dose interaction effect, Greenhouse-Geisser ε = *0.65, F(2.6, 78.5) = 8.87, p < 0.001)*, showing a significantly different dose-response effect for guanfacine in SHRs vs. WKYs. There was also a Strain x Dose x Within-Session Segment interaction effect, *Rao R Form 2 (4, 27) = 4.643, p < 0.006*. Follow-up t-tests for independent samples showed that there was no significant difference between the control WKYs following placebo and the SHRs following the 0.6 mg/kg dose *t(30) = 1.927 p > 0.06, two-tailed test*. There were significant differences between the SHRs and the WKY following placebo for all the other doses. Therefore, the 0.6 mg/kg dose was the only one that apparently normalized SHR behavior.

#### Impulsiveness

**Figure 3:**
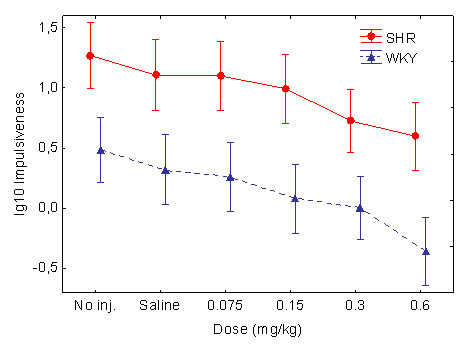
Effect of the different guanfacine doses on impulsiveness (lg10 transformed), responding within 0.67 s following the previous lever press, in SHR and WKY. Means ± 95% confidence intervals.

**Figure 4:**
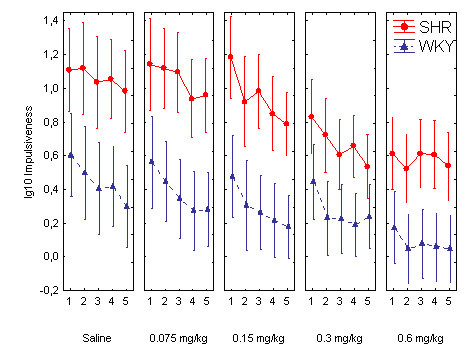
Effect of the different guanfacine doses on impulsiveness (lg10 transformed) across the five 18-min within-session segments in SHR and WKY. Means ± 95% confidence intervals.

The SHRs were significantly more impulsive than the WKY controls (Figures 3 and 4). There was a drift in the baseline of the SHR strain. The statistical effects of the drift were reduced by lg10-transformations.

Impulsiveness was reduced by guanfacine in a dose-related fashion in both strains. The ANOVA showed main effects of strain, *F(1, 30) = 18.51, p < 0.001*; dose, Greenhouse-Geisser *ε = 0.61, F(2.4, 73.1) = 23.5, p < 0.001*; and within-session segment, Greenhouse-Geisser *ε = 0.57, F(2.3, 68.3) = 24.2, p < 0.001*. There was no interaction effect involving both the strain and dose variables, because the drug appeared to have a similar effect in the WKYs as in the SHRs. Follow-up t-tests for independent samples showed that there was no significant difference between the control WKYs following placebo and the SHRs following the 0.3 and the 0.6 mg/kg doses: *t(30) < 1.69 p > 0.10, two-tailed tests*, although there were significant differences between the WKYs following placebo and the SHRs for the other doses. Therefore, the 0.3 and 0.6 mg/kg doses apparently normalized SHR behavior.

#### Sustained attention

**Figure 5:**
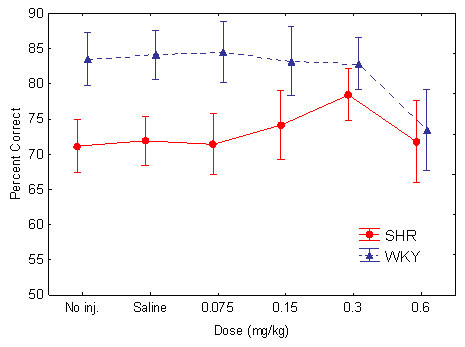
Effect of the different guanfacine doses on sustained attention, percent choice of the correct lever in SHR and WKY. Means ± 95% confidence intervals.

**Figure 6:**
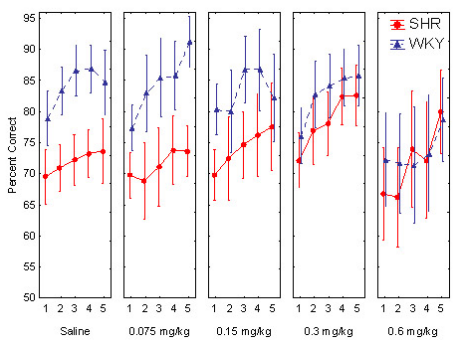
Effect of the different guanfacine doses on sustained attention, percent choice of the correct lever across the five 18-min within-session segments in SHR and WKY. Means ± 95% confidence intervals.

The sustained attention behavior improved throughout the study as well as within session in both strains although the SHR showed a consistently poorer behavior than the WKY controls (Figures 5 and 6).

Without active drug, the SHR had a lower percent correct lever choice, i.e., poorer sustained attention, than WKY controls. Guanfacine improved the poor performance of the SHR (Figure 5). The effect was more pronounced towards the end of the session (Figure 6). The ANOVA showed main effects of strain, *F(1, 30) = 12.4, p < 0.002*; dose, Greenhouse-Geisser *ε = 0.47, F(1.9, 57.0) = 6.3, p < 0.004*; and within-session segment, Greenhouse-Geisser *ε = 0.84, F(3.4, 100.6) = 21.1, p < 0.001*. These effects show that the SHRs were consistently poorer than the WKY controls and that both strains' behavior improved towards the end of the 90-min sessions. There was also a Strain x Dose interaction effect, which shows that there was a dose-related improvement in the SHR following the drug, but not in the WKY controls, Greenhouse-Geisser ε *= 0.47, F(1.9, 57.0) = 4.32, p < 0.02)*. There was no Strain x Dose x Within-Session Segment interaction effect. Follow-up t-tests for independent samples showed that there were significant differences between the WKYs following placebo and the SHRs following all the doses, although the 0.3 mg/kg dose came closest to normalizing SHR behavior: *t(30) = 2.129 p = 0.042, two-tailed test*.

## Discussion

The main clinical symptoms of ADHD are inattentiveness, overactivity, and impulsiveness [1]. The best validated animal model of ADHD, the SHR, showed pronounced impulsiveness, overactivity, and reduced sustained attention during baseline conditions, and all three measures were improved by treatment with guanfacine.

Sedation following guanfacine is reported clinically [12,13]. Sedation could possibly explain the reduction in SHR impulsiveness as the dose-response curves were similar in both the WKYs and SHRs. It seems less likely, however, that sedation can explain the reduction in hyperactivity where the dose response curve was clearly different in the SHRs and WKYs; and highly unlikely to explain the improvement in sustained attention in the SHR where a decrease would be a more likely consequence of sedative activity.

Without more data on the specificity of higher doses of guanfacine, it cannot be concluded that only norepinephrine is affected by the doses used presently. It might be that improved sustained attention is due to improved noradrenergic functioning following low doses and that the higher doses reducing overactivity and impulsiveness may involve other neuromodulators like dopamine in addition to norepinephrine.

In conclusion, the present results support the beneficial effects of guanfacine on ADHD behavior reported clinically [12,13] and experimentally in primate models of frontal function [14,15,23-25]. It is likely that guanfacine improved prefrontal functions in the SHR, cf. [14,15]. It cannot be concluded, however, that the effects of the drug are mediated solely by norepinephrine.

## Supplementary Material

Additional file 1 WKYm.wmv. The video shows a normal male WKY control rat performing the visual discrimination task. Click here for file

Additional file 2 SHRm.wmv. The video shows a Spontaneously Hypertensive Rat (SHR) performing the visual discrimination task. The rat is overactive and inattentive. Click here for file
